# Semi-supervised wildfire smoke detection based on smoke-aware consistency

**DOI:** 10.3389/fpls.2022.980425

**Published:** 2022-11-08

**Authors:** Chuansheng Wang, Antoni Grau, Edmundo Guerra, Zhiguo Shen, Jinxing Hu, Haoyi Fan

**Affiliations:** ^1^ Department of Automatic Control Technical, Polytechnic University of Catalonia, Barcelona, Spain; ^2^ Henan Academy of Forestry, Zhengzhou, Henan, China; ^3^ Shenzhen Institute of Advanced Technology, Chinese Academy of Sciences, Shenzhen, China; ^4^ School of Computer and Artificial Intelligence, Zhengzhou University, Zhengzhou, China

**Keywords:** wildfire smoke detection, semi-supervised learning, smoke-aware consistency, triple classification assistance, smoke detection network

## Abstract

The semi-transparency property of smoke integrates it highly with the background contextual information in the image, which results in great visual differences in different areas. In addition, the limited annotation of smoke images from real forest scenarios brings more challenges for model training. In this paper, we design a semi-supervised learning strategy, named smoke-aware consistency (SAC), to maintain pixel and context perceptual consistency in different backgrounds. Furthermore, we propose a smoke detection strategy with triple classification assistance for smoke and smoke-like object discrimination. Finally, we simplified the LFNet fire-smoke detection network to LFNet-v2, due to the proposed SAC and triple classification assistance that can perform the functions of some specific module. The extensive experiments validate that the proposed method significantly outperforms state-of-the-art object detection algorithms on wildfire smoke datasets and achieves satisfactory performance under challenging weather conditions.

## 1 Introduction

Failure to detect and control wildfire in a timely manner can result in devastating disasters to forests (Ray et al., 2017; Niccoli et al., 2019). Therefore, it is very important that forest safety monitoring systems are able to detect fire and smoke in a timely and effective manner (Barmpoutis et al., 2014). Early research into fire monitoring systems mainly focused on the detection of flames. However, smoke detection is more suitable than fire detection for forest monitoring systems because fires develop slowly in the early stages and are not easily detected by cameras. As a result, fire detection-based security monitoring systems do not provide alerts in time compared to smoke detection-based security monitoring systems. Therefore, smoke detection is more suitable than fire detection for the role of fire monitoring algorithms in forest scenarios (Filonenko et al., 2017). Sensor-based smoke detectors rely on smoke ionization to produce particulate matter and then perform smoke detection (Adamian et al., 1996). This principle means that the sensor-based smoke detector can only achieve good performance in small scale scenarios, but it cannot be applied to forests with large areas and complex landscapes like forests (Lin et al., 2018). To solve this problem, many researchers have carried out studies on computer vision-based smoke detection algorithms (Dimitropoulos et al., 2016). Earlier smoke detection algorithms could only determine the presence of smoke in a scene and could not localize it (Miyazawa, 2002). However, precise localization of smoke areas can help fire-fighting systems to provide more accurate alerts. Therefore, the accurate localization of smoke has become an important issue in the field of computer vision in recent years (Jia et al., 2016). In this article, we will refer to this as smoke detection.

Most of the early vision-based smoke detection was based on inference algorithms with shallow feature representations (Kaabi et al., 2017). These methods use visual features to represent smoke, such as color, speed, transparency and direction (Barmpoutis et al., 2014). However, current smoke detection algorithms based on feature representations still suffer from some shortcomings due to the lack of robust mechanisms for characterizing smoke motion and external morphology (Jia et al., 2019), which means that the performance will decline significantly when the running environment changes. Therefore, these kinds of smoke detection methods still need further improvement in terms of generalization and interference (Yuan et al., 2019).

**Figure 1:**
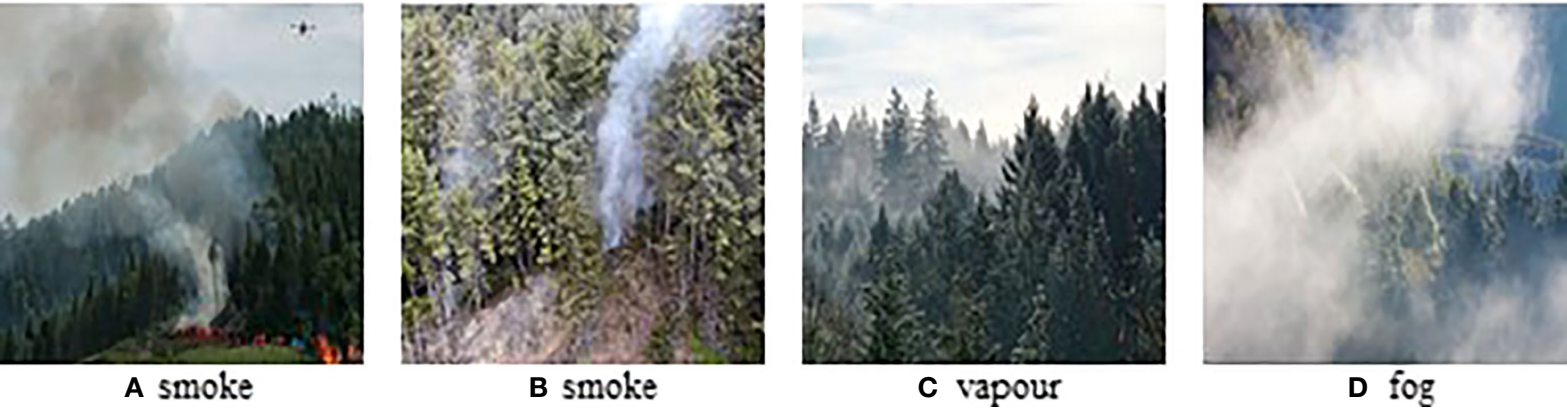
Some challenging images. **(A, B)** are smoke images; **(C, D)** are vapour and fog images.

With the development of artificial intelligence in industry, agriculture and forestry (Chen et al., 2017; Ferrag et al., 2017; Ray et al., 2017; Zhu et al., 2018; Ferrag et al., 2020; Liu et al., 2020), many researchers have begun to focus on deep learning-based smoke detection algorithms, while the performances remain unsatisfactory for the following reasons: 1) The shape of the smoke is constantly changing and its visual characteristics are susceptible to be influenced by the background; 2) Smoke has different visual features at different stages of combustion, so it is difficult for CNNs to learn high-dimensional features of smoke that are adapted to different stages of combustion; 3) As shown in **Figure 1**, some of the objects that appear in the forest have an appearance similar to that of smoke, which makes the model susceptible to misinterpretation of these normal conditions in practical applications. Therefore, a good wildfire smoke detection algorithm needs to be able to accurately detect smoke regions in the complex environments.

Combining the above issues with the current problems in the field of smoke detection, this paper proposes SAC for the following reasons: 1) the traditional learning-based object detection algorithms are usually dedicated to the design of the model structure, ignoring the impact of the training data on performance in different scenarios. 2) traditional object detection algorithms are designed to working in clear scenes by default. However, in real-world applications, the images captured by camera are often affected by unusual weather conditions, resulting in reduced detection accuracy. 3) traditional fully supervised object detection relies to some extent on over-fitting of background information. Thus, when the running scenario changes significantly, the performance of the model will be severely degraded.

In order to address the above issues, the following contributions have been made in this paper:

- In this paper, a new SAC method is proposed to solve the problem of insufficient amount of training data. Specifically, we first obtain pseudo-labels for the unlabeled data by using a preliminary model trained on the labelled data. The original unlabeled image was then cropped into eight patches based on pseudo-labelling, and eight different data augmentation methods were then randomly applied to these patches. The next step is to make the model’s detection on the patch consistent with the pseudo label by back propagation. The experimental results show that the proposed SAC can effectively improve the detection accuracy as well as the robustness of the model.
- In this paper we optimized the traditional fully supervised LFNet model for fire smoke detection and proposed a simplified LFNet-v2. Specifically, we remove the multi-scale feature extraction module and attention mechanism used in LFNet due to the proposed SAC method and triple classification assistance can help the model to better understand the smoke and background. In addition, the removal of these two modules could improve the inference speed of LFNet-v2.
- In order to avoid the disturbance of smoke in forest scene by objects with similar visual features, a triple classification assistance is proposed. Specifically, in the training phase, the proposed method adds the sky class as a detection class compared to the traditional smoke detection. This training strategy can help the model better recognize the smoke and the background.

The rest of this article is arranged as follows. Related work on traditional fire monitoring methods and learning-based fire monitoring methods are given in Section 2.1 and Section 2.2 respectively. The proposed method is introduced in Section 3. The comparison and ablation experimental results are introduced in Section 4. The conclusion is drawn in the Section 5.

## 2 Related Work

Early work in the computer vision community on smoke monitoring focused on smoke detection based on visual features, but this approach tended to have significant false positives and negatives. Recently, learning-based smoke detection methods have evolved significantly with the increase in computing power. In order to describe the progress made in the field of artificial intelligence in terms of fire and smoke detection algorithms, this section analyzes the relevant literature from two different perspectives, namely traditional machine learning-based and modern deep learning-based fire monitoring algorithms respectively.

### 2.1 Traditional fire monitoring methods

Early research into smoke detection focused on the underlying visual features of the image. For example, Chen et al. simultaneously used the RGB and HIS color spaces to studying the dynamic characteristics of the smoke (Chen et al., 2004). Marbach et al. studied the YUV color space at the pixel level and used it to determine whether a fire was occurring in the current scene (Marbach et al., 2006). Celik et al. proposed a fire detection algorithm based on the pixel-level YCbCr color space, and also put forward a new rule for distinguishing chromaticity and brightness (Celik and Demirel, 2009). Habiboğlu et al. proposed a real-time fire detector based on SVM (Habiboğlu et al., 2012).

The smoke detection methods based on color representation are susceptible to brightness and are poorly robust to changes in the environment (Chaturvedi et al., 2022). In recent years, more and more researchers have been using different characterization methods for smoke detection of fires. Among these works, Borges et al. combined color, texture and roughness with the Bayesian classifier to recognize the fire and smoke (Borges and Izquierdo, 2010). Toreyin et al. adopted spatiotemporal wavelet analysis to detect the areas of fire in the video (Töreyin et al., 2006). Di Lascio combined the color and motion information in the video to detect the fire (Lascio et al., 2014). Dimitropoulos et al. adopted the spatiotemporal features for fire detection, and then used SVM to classify the candidate regions (Dimitropoulos et al., 2014). Even though these methods can improve the performance of the model, they often have poor robustness and generalization abilities. To address this problem, many researchers have begun to focus on deep learning-based smoke detection methods.

### 2.2 Deep learning-based fire monitoring methods

Recently, deep learning has gradually replaced machine learning as a mainstream approach to fire and smoke detection (LeCun et al., 2015). Based on SqueezeNet (Iandola et al., 2016), Khan et al. proposed a lightweight fire detection algorithm, which can locate and identify objects simultaneously (Muhammad et al., 2018). This method can balance fire detection accuracy and inference speed well with few parameters. Yin et al. adopted a deep normalized CNN to speed up training and improve the performance of the smoke detection (Yin et al., 2017). Zhang et al. used both real and synthetic smoke images in their training set (Zhang et al., 2018). However, the experimental results show that these methods cannot solve the problem of insufficient training dataset.

Learning-based detection methods can automatically extract features that are beneficial for smoke detection. However, the performance of these methods will be severely degraded by the lack of training data for forest scenes due to the inherent disadvantages of the fully supervised training strategy. To address this problem, this paper proposes a semi-supervised smoke detection method that allows the model to achieve high accuracy despite using insufficient data. The specific details of the proposed method are described in detail in the next section.

## 3 Methodology

**Figure 2:**
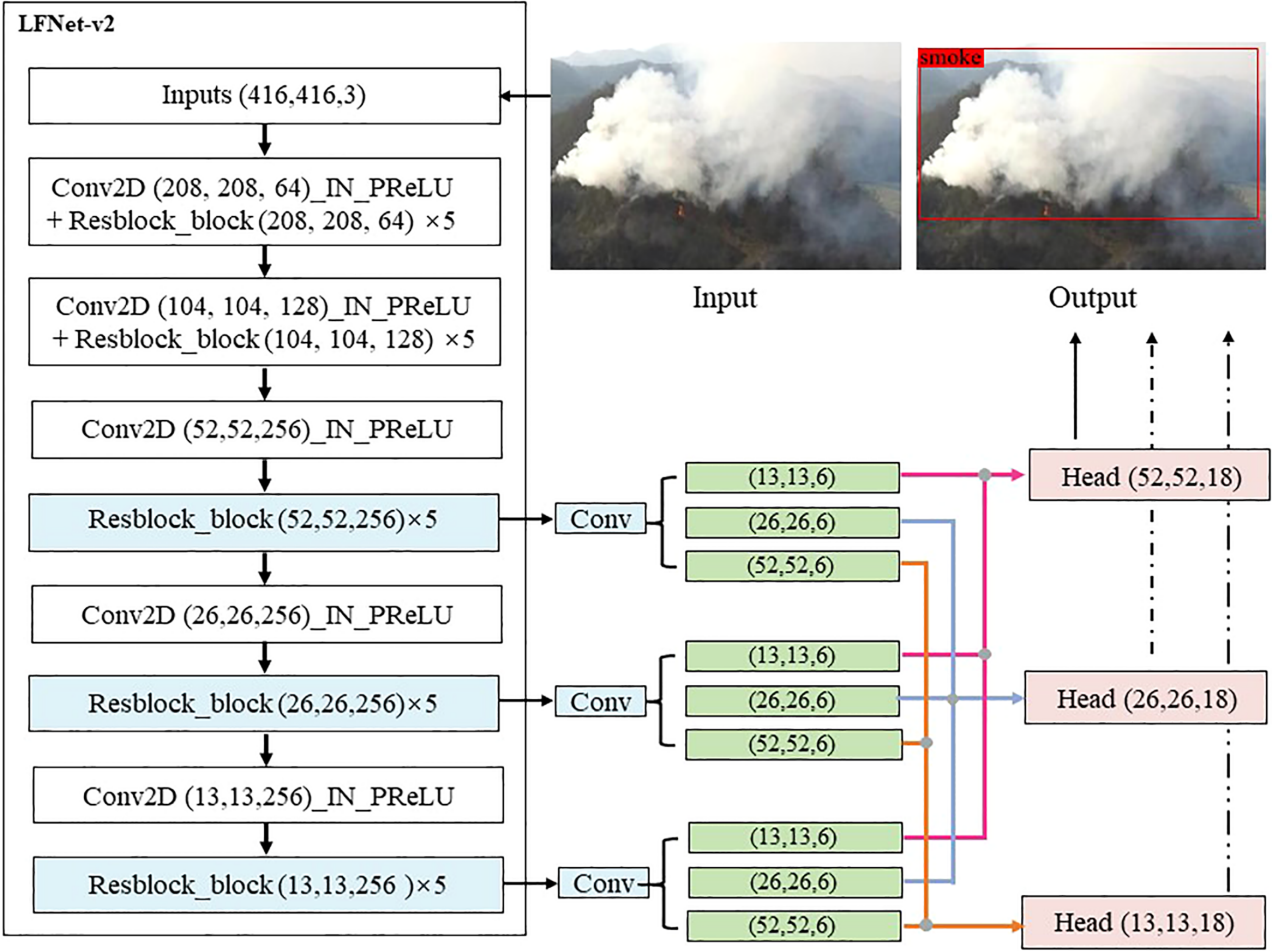
The framework of the proposed LFNet-v2.

The structure of the LFNet-v2 is shown in **Figure 2**. Since the SAC and triple classification assistance proposed in this paper can provide the functionality of some special modules applied in LFNet to a certain extent, this paper simplifies the classical fire and smoke detection model LFNet (Shen et al., 2020) and proposes LFNet-v2. Specifically, we removed the multiscale feature extraction module from LFNet because the SAC strategy proposed in this paper allows the network to adapt to different contexts. In addition, we also removed the attention mechanism that plays an important role in LFNet for the same reason. Lastly, since our work focuses on smoke detection only, we discard the original loss function *SCP* (*x*) designed specifically for the fire detection, and merely use the loss function employed in YOLOv3 (Redmon and Farhadi, 2018) for LFNet-v2.

### 3.1 The framework of LFNet-v2

The structure of LFNet-v2 is shown in **Figure 2**. Similar to YOLOv3, LFNet-v2 first normalizes the size of the input image to 416 * 416 * 3 by using a uniform grey fill box, and then halves the image size by Conv2D, which has a convolution kernel of 3, a step size of 2 and a padding of 1. Finally, an input image of size (416, 416, 3) will eventually be transformed by LFNet-v2 into a high-dimensional feature map of size (13, 13, 256). One important characteristic of LFNet-v2 is the incorporation of a residual block (He et al., 2016). The advantages of residual network is easiness for optimization, which can also improve accuracy by increasing network depth. Internal skip connections are adopted in the residual block, which can deal with the gradient vanishing problem caused by the increasing depth in the depth of the neural network. A residual block with kernel size 3 and step size 2 is first run for LFNet-v2. We perform this convolution at this feature layer and add the results to LAYER. As a result, the network structure of LFNet-v2 can be deepened considerably.

The PReLU (He et al., 2015) is used for each convolution of LFNet-v2. After convolution, each part will be normalized using Instance Normalization (IN) (Ulyanov et al., 2016), and then PReLU will be employed. A common ReLU sets all negative values equal to zero, whereas a nonzero slope is assigned by PReLU to all negative ones, and its mathematical expression is:


(1) $$f\;\left(y_{i}\right)\;=\;\{\begin{array}{ll} y_{i}, & \text{if }y_{i}>0\\ a_{i}y_{i}, & \text{ if }y_{i}\leq 0 \end{array}$$

In the last step, convolution is performed to the Resblocks of the 6th, 8th and 10th layers of the network, and the convolution blocks of size (13,13,6), (26,26,6) and (52,52,6) are output respectively. Lastly, features of uniform size are spliced together by feature splicing, and the heads of three different sizes of (52,52,18), (26,26,18) and (13,13,18) are obtained, which are used to detect large, medium and small size of smoke, respectively.

#### 3.1.1. Loss function

The task of the LFNet-v2 is the accurate localization of the smoke region. Therefore, the loss function in YOLOv3 is used directly in this paper, which consists of object location offset loss, classification loss and target confidence loss as follow:


(2) $$L\;\left(o,\;c,\;O,\;C,\;l,\;g\right)\;=\;\lambda_{1}L_{conf}\;\left(o,\;c\right)\;+\;\lambda_{2}L_{cla}\;\left(O,\;C\right)\;+\;\lambda_{3}L_{loc}\;\left(l,\;g\right)$$

where, *λ*_1_, *λ*_2_ and *λ*_3_ are the balance coefficients, *L_conf_* (*o*, *c*) is the confidence loss, *L_cla_* (*O*, *C*) is the classification loss, and *L_loc_* (*l*, *g*) is the localization loss. In this paper, We set *λ*_1_, *λ*_2_ and *λ*_3_ to 1, 0.5 and 1 respectively. Notice that since an auxiliary multi-class strategy is used to enable the model to better extract scene information in this paper, the classification loss is retained in our model. It should be emphasised that the design of the loss function is not the focus of this paper, but the model proposed in this paper can still achieve better results using this underlying loss function.

### 3.2 Smoke-aware consistency

Methods that can maintain consistency between image features under perturbation have achieved good performance in semi-supervised learning (Tarvainen and Valpola, 2017). On the other hand, as the surface features of smoke images are not very distinct and there is no fixed paradigm for the shape and color of smoke, it is difficult to perturb the network on the extracted smoke image features by applying simple data augmentations to the input image. Furthermore, one reason for the apparent discrepancy in detection results obtained on different images is that the model was over-fitted to the limited training data, which made the model overly dependent on background information when extracting smoke features. This means that although the model achieves consistency in low-dimensional augmentation, it still fails to produce a consistent embedding distribution across content. In addition, one reason for the significant variation in features across backgrounds is that the model over-adapts to the limited training data, resulting in features that are too dependent on contextual cues and not sufficiently self-aware. One way to deal with this problem is to generate more robust features by maintaining consistency between features across contexts, which can also alleviate the over-fitting problem to some extent. Inspired by this, we propose the SAC method, which is a novel and effective semi-supervised training strategy. Experimental results show that the proposed method outperforms general data augmentation methods.

**Figure 3:**
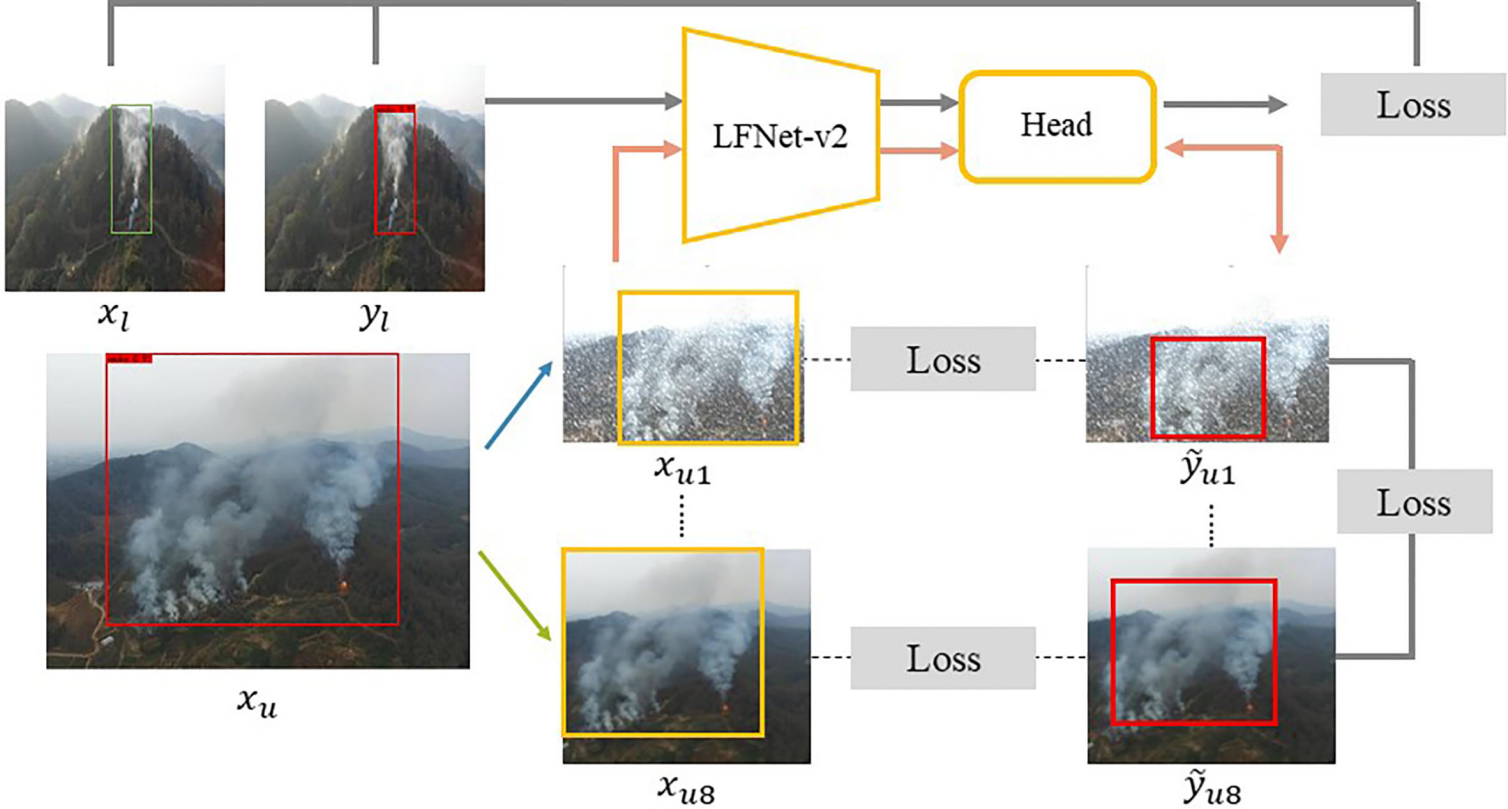
Overview of the proposed SAC.

**Figure 3** shows the semi-supervised training framework used in this paper. Specifically, there are two groups of different inputs, which are *x_l_* and *x_u_*, representing the labeled and unlabeled data respectively. The labeled image *x_l_* is input to the encoder network *ε* to obtain the feature map *f_l_* = *ε*(*x_l_*); then, the detection head *H* obtains prediction result *p_l_* = *H*(*fl*); finally, it is supervised by the ground truth label *y_l_* for back propagation. The model can obtain certain smoke detection ability using the labeled data, which can help the unlabeled data to obtain the pseudo-label *ỹ_o_*.

**Figure 4:**
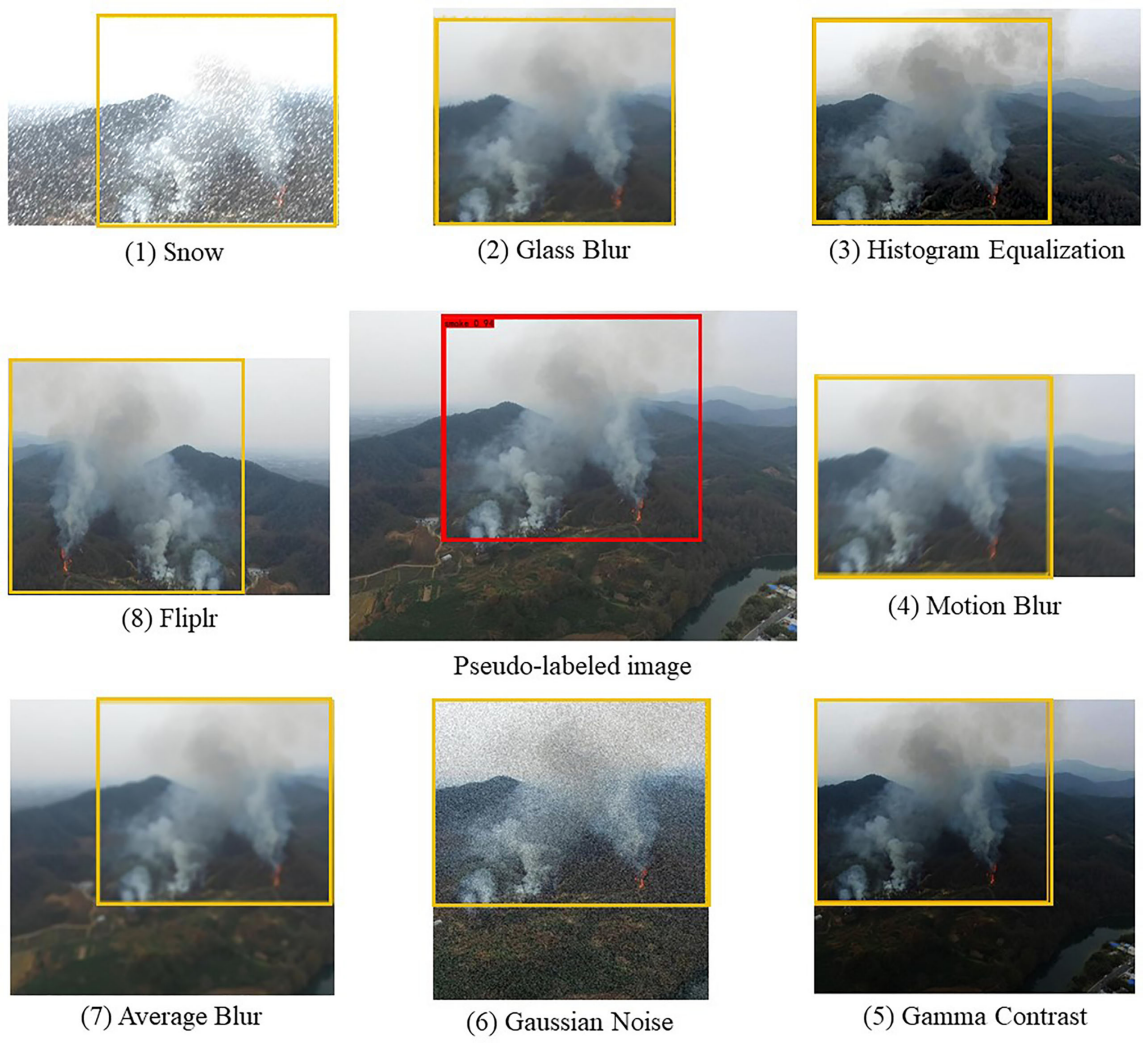
Examples of data augmentation and patch interception schemes used in the SAC.

For the unlabeled image data *x_u_*, eight different patches are obtained based on the pseudo-label *ỹ_o_* by random matting in eight different directions on the same overlapping area. Then, one of eight different image augmentations is chosen randomly and performed to one of these eight different patches, and these augmentations are: 1. Glass Blur; 2. Histogram Equalization; 3. Motion Blur; 4. Gamma Contrast; 5. Gaussian Noise; 6. Average Blur; 7. Fliplr; 8. Snow. The example of image augmentation and image matting are shown in **Figure 4**.

Subgraphs of unlabeled image data after low dimensional data augmentation are then fed into LFNet-v2 for smoke detection. Notice that since LFNet-v2 is a model trained using a small amount of labeled data, the model detection accuracy is not very reliable. Therefore, this paper proposes to improve the detection performance of LFNet-v2 on unlabeled data by following two supervised methods. First, it is required that the pseudo-labelling of *x_u_* in the perturbed cropping region is back-propagated between the detection result of *x_u_* and *ỹ_u_* by the formula (2); second, it is required that all subgraphs between *ỹ_u_*_1_ and *ỹ_u_*_8_ are back-propagated by the formula (2).

In the meantime, the availability of pseudo-labels *ỹ_o_* for unlabelled data *x_u_* cannot be determined as the shape of smoke produces different visual features during drifting, which would pose a significant challenge for fire and smoke detection where training data is scarce. To address this problem, we set the offset coefficient *μ* of the pseudo-labels according to the detection accuracy achieved on the labeled validation set, so that the pseudo-labels will drift in a certain random direction according to the coefficient *μ*. As the detection accuracy decreases, the larger the scale in which the pseudo-labels drift.

During the experiment, the performances of the proposed SAC method in this paper were proved *via* specific comparison and ablation experiments. The proposed SAC framework is optimized following **Algorithm 1**.

Algorithm 1. SMOKE-AWARE CONSISTENCY 
Input: labeled & unlabeled images ∈ Dataset
Output: detection results
1. *N*1 ← 30
2. *N*2 ← 30
3. *i* ← *counter* ← 0
*4.* **
*For*
** *i 0 to N1 by 1* **
*do*
**
*5. Loss*(*LFNet – v*2(*images*), *label 6. lterative 7. Optimization*
8. **End for**
*9. i* ← *counter* ← 0
*10.* **
*For*
** *i 0 to N2 by 1* **
*do*
**
*11. pseudo label* ← *LFNet – v*2(*images*)
*12. cropped area* ← *LFNet – v*2(*augmentation*(*cropped area*))
*13. Loss*(*pseudo label*, *LFNet – v*2 (*cropped area*))
*14. Loss*(*LFNet – v*2 (*cropped area*), *LFNet – v*2 (*cropped area*))
*15. lterative 16. Optimization*
17. **End for**
18. **Return** 0;

### 3.3 Triple classification assistance

**Figure 1** indicates that forest smoke is often easily confused with objects in the background, such as clouds and the sky. For the model to better distinguish the background and smoke region, this paper proposes a triple classification assistance strategy. Specifically, images of smoke in forests fall into three categories: background, smoke and sky. Extensive experiments show that the proposed triple classification assistance can help the model distinguish the image features that are easily confused with the smoke features and improve the model’s detection performance. We compared the performance of this model with or without classification assistance to verify its practical value for forest scene smoke detection.

## 4 Experiments

To verify the superiority of the proposed method, this section compares it with other state-of-the-art algorithms for classification and detection of smoke images. In addition, we have carried out sufficient ablation experiments to prove the practical value of the innovation proposed in this paper. All the comparison algorithms train a total of 60 epochs and performed on a server with Intel (R) Core (TM) i7-8750H CPU 10720GHz, 16.0GB RAM, and NVIDIA 1070. The deep learning framework used to train these algorithms is PyTorch 1.7.

### 4.1 Dataset

In order to prove that the proposed method can provide stable performances under suboptimal imaging conditions, we classify images into the following categories by refer to the method in (Khan et al., 2019): 1) Smoke; 2) Smoke with fog; 3) Non-smoke; 4) Non-smoke with fog. It is worth noting that the method proposed in (Khan et al., 2019) is a smoke recognition algorithm for the haze weather only, but the proposed method is applicable to any specific conditions. For example, the experimental results show that the proposed algorithm has good performance in foggy forest scene. In this case, the smoke images for foggy days are synthesized using the atmospheric scattering model (McCartney, 1977). The mathematical equation for the atmospheric scattering model is as follows:


(3) I (x) = J (x) t (x) + α (1 − t (x))

Where *I*(*x*) is the haze-degraded image, *J*(*x*) is the haze-free scene, *α* is the global atmospheric light representing the ambient light in the atmosphere, and *t*(*x*) is the transmission of the intrinsic luminance in the atmosphere. In this paper, we set *α* and *t*(*x*) to 0.7 and 0.5, respectively.

**Figure 5:**
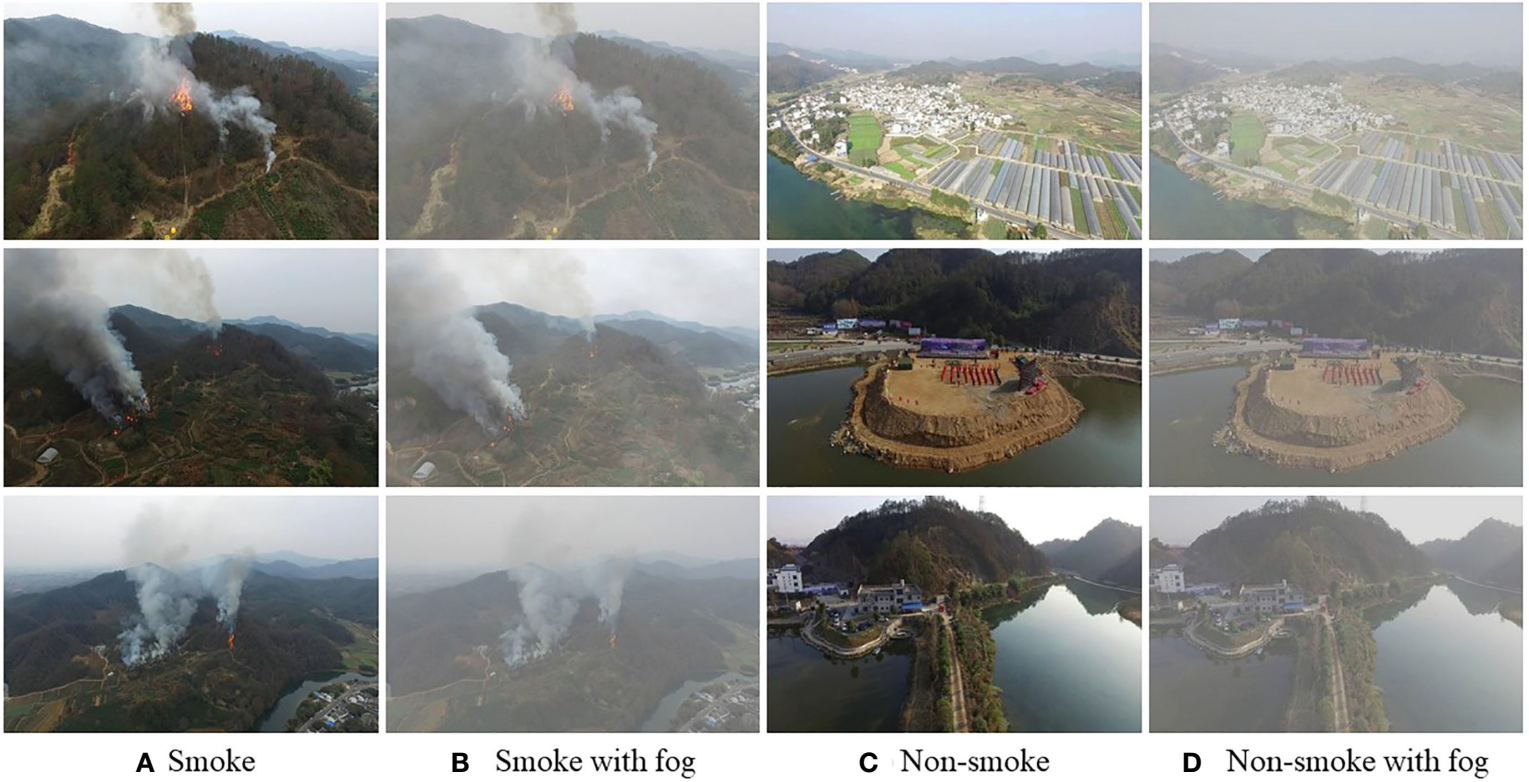
Representative images of smoke, smoke with fog, non-smoke, non-smoke with fog.

As shown in **Table 1**, the data set used in this paper includes 4,014 images, 50% of which are synthetic foggy images, and the rest 50% are the original images. We use fully supervised smoke detection (FSSD) and semi-supervised smoke detection (SSSD) methods as comparison algorithms to demonstrate the superiority of the proposed method. **Table 1** details the distribution of the dataset, and **Figure 5** shows some images of the dataset used in this paper. For fully supervised learning, 60% labeled images are used for training, 20% labeled images for verification and 20% unlabeled images are used for testing. For semi-supervised learning, 30% labeled images and 50% unlabeled images are used for training, and 50% unlabeled images are used for testing. But, the test sets of FSSD and SSSD are consistent. Many smoke detection algorithms can only determine the presence or absence of smoke in the current scene, but cannot accurately locate areas of smoke. Therefore, in order to compare algorithms that can only accomplish smoke image classification as comparison algorithms as well. We evaluate the proposed method from two perspectives, image classification and object detection.

**Table 1:** Overall statistics of training, validation, and testing data for the proposed system.

FSSD SSSD	Total		Non-smoke		Smoke		Non-Smoke With fog		Smoke With fog	
Total	4014		1546		461		1546		461	
Training Data	2406	1202	927	463	276	138	927	463	276	138
Validation/Semi Data	802	2006	309	773	92	230	309	773	92	229
Testing Data	806	806	310	310	93	93	310	310	93	94

### 4.2 Smoke classification

Some security systems only need the model to determine if there is smoke in the current scene and do not need to obtain the exact location of the smoke area. Therefore, image classification methods are sufficient to meet the requirements of such systems. To demonstrate the image classification capability of the proposed model, we set the confidence level of smoke detection to 0.4 and then compare this method with other state-of-the-art image classification algorithms.

Comparison algorithms can be classified into three different types: the first type is the fully supervised image classification algorithm applied to non-specific scenes, the second type is fully supervised images classification algorithms specifically designed for smoke image classification, and the third type is common semi-supervised image classification algorithms. Specifically, the fully supervised image classification algorithms for non-specific scenes include mobileNet (Howard et al., 2017), ResNet18 (He et al., 2016) and VGG16 (Simonyan and Zisserman, 2014); the fully supervised smoke image classification algorithms include DCNN (Gu et al., 2019), SIUM (Yu et al., 2019) and DarkCDCN (Liu et al., 2019); the semi-supervised image classification algorithms include SESEMI (Tran, 2019) and SRC-MT (Liu et al., 2020).

**Figure 6:**
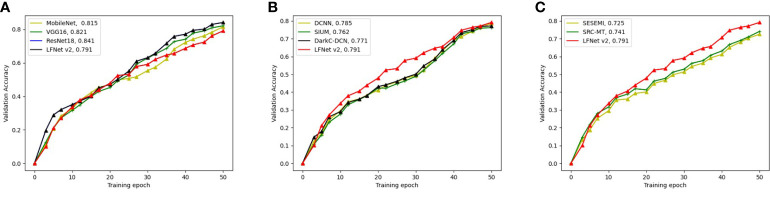
Study on the performance of wildfire scene image classification algorithm. **(A)** denotes the classification accuracy of the commonly used superintendency image classification algorithm; **(B)** denotes the classification accuracy of the commonly used superintendency image classification algorithm; and **(C)** denotes the classification accuracy of the commonly used semi-superintendency image classification algorithm.

**Figure 6** represents the training process and the final classification accuracies of each model. Specifically, **Figure 6A** shows that MobileNet is a fully supervised image classification algorithm with an accuracy of 81.5%, and the accuracies of VGG16 and ResNet18 are 82.1% and 84.1%, respectively. Therefore, ResNet18 achieved the best performance among all fully supervised algorithms for non-specific scenes. Although the proposed method only achieved an accuracy of 79.1%, it was only trained on 30% labeled data. In contrast, MobileNet, VGG16 and ResNet18 were subjected to 80% of the labeled data. Nevertheless, the accuracy of the LFNet-v2 was merely 5% lower than that of ResNet18, which shows that the proposed LFNet-v2 still managed to achieve great classification performance with insufficient training data.

**Figure 6B** shows that LFNet-v2 outperformed the other fully supervised smoke detection algorithms, with a classification accuracy of 79.1%. The classification accuracies of the algorithms for comparison are as follows: the DCNN (Gu et al., 2019) had an accuracy of 78.5%, SIUM (Yu et al., 2019) achieved an accuracy of 76.2%, and the accuracy of DarkC-DCN (Liu et al., 2019) was 77.1%. The classification accuracy of LFNet-v2 was 0.6%, 2.9% and 2.0% higher than that of DCNN, SIUM and DarkC-DCN, respectively. Experiments have shown that smoke image classification algorithms that are not specifically designed for forest scenes do not provide accurate classification when applied to forest scenes.

**Figure 6C** demonstrates the performances of two classic semi-supervised image classification algorithms, i.e., SESEMI (Tran, 2019) and SRC-MT (Liu et al., 2020), which achieved the classification accuracies of 72.5% and74.1%. LFNet-v2 had an accuracy of 79.1%, 6.6% and 5% higher than that of SESEMI and SRC-MT, respectively.

In summary, **Figure 6** shows that although the classic semi-supervised image classification algorithms can generally achieve relatively good performances on the smoke images with forest scenes, these models cannot achieve very high accuracy because there is no special module designed for such forest scene. However, the proposed algorithm achieves better classification results than the usual semi-supervised image classification algorithms.

**Table 2** describes the performance of smoke detection algorithms based on image classification in more detail. As can be seen from the **Table 2**, the proposed LFNet-v2 performs better in fog than other comparison algorithms. Specifically, in the foggy non-smoke scene, the classification accuracy of the proposed model was as higher as 73.4%, which was the second highest among all algorithms for comparison, only next to ResNet18 with a classification accuracy of 74.3%. In the foggy smoke scenes, the accuracy of the proposed method was 91.2%, which was the highest among various algorithms. In contrast, the accuracies of ResNet18 and SIUM were both 82.1%, which was in the second place. Therefore, the proposed method is more suitable for classification of degraded images under sub-optimal imaging conditions. However, for the clear non-smoke forest scenes, other algorithms for comparison slightly outperformed the algorithm proposed in this paper. Specifically, the detection accuracy of the algorithm was only 90.1% for obvious smoke scene, 8.3% lower than the SRC-MT maximum.

**Table 2:** Comparison of the proposed method with eight state-of-the-art image classification methods.

Networks		MoblieNet	ResNet18	VGG16	DCNN	SIUM	DarkC- DCN	SESEMI	SRC-MT	LFNet-v2
Foggy	Non-smoke	71.9%	74.3%	69.3%	67.8%	66.9%	68.1%	61.1%	60.4%	73.4%
	smoke	77.1%	82.1%	82.8%	74.8%	82.1%	75.5%	77.2%	69.5%	91.2%
	Total	73.1%	76.1%	72.4%	69.4%	70.4%	69.8%	64.8	62.5%	77.5%
Clear	Non-smoke	79.1%	82.2%	94.9%	85.6%	80.6%	81.9%	78.9%	81.9%	77.9%
	smoke	84.5%	91.2%	95.6%	94.3%	86.7%	92.8%	84.6%	98.4%	90.1%
	Total	89.9%	88.1%	98.8%	87.6%	82.0%	84.4%	80.2%	85.7%	80.7%
Total		81.5%	82.1%	84.1%	78.5%	76.2%	77.1%	72.5%	74.1%	79.1%

**Table 2** shows that the proposed method achieves a classification accuracy of only 79.1%, ranking fourth among all comparison algorithms. However, it is worth noting that the top three algorithms are fully supervised detection algorithms with 80% of the labelled training data, while the proposed algorithm are trained using only 30% of the labeled data. However, the classification accuracy of this algorithm is still 6.6% and 5% higher than the other two semi-supervised image classification algorithms.

### 4.3 Smoke detection

Advanced intelligent fire-fighting robots need to accurately locate areas of smoke in order to complete a series of fire-fighting instructions (Park et al., 2019). To the best of our knowledge, this paper is the first semi-supervised smoke detection algorithm. Therefore, the proposed method is compared with the general fully supervised smoke detection algorithms, general fully supervised object detection algorithms and general semi-supervised object detection algorithms to be used to demonstrate the superiority of the algorithm proposed in this paper. Specifically, the fully supervised object detection algorithms include the Faster RCNN (Ren et al., 2015), CenterNet (Zhou et al., 2019), and YOLOx (Ge et al., 2021); the fully supervised smoke detection algorithms include the DSATA (Zhao et al., 2020), Frizzi et al. (2016), and 3DCNN (Lin et al., 2019); the semi-supervised object detection algorithms are the Soft-Teacher (Xu et al., 2021) and STAC (Sohn et al., 2020). Although many excellent evaluation strategies have been proposed recently (Rodríguez-Fdez et al., 2015), we still choose the most classic COCO criteria as our evaluation strategy, including AP (averaged average precision over different IoU thresholds, the primary evaluation metric of COCO), *AP*_50_ (average precision for IoU threshold 0.50), *AP*_75_ (average precision for IoU threshold 0.75), *AP_S_* (AP for small objects), *AP_M_* (AP for medium objects), and *AP_L_* (AP for large objects).

The smoke detection results are shown in **Table 3**. The proposed LFNet-v2 achieved the highest mAP of 0.452, meaning that it had the best overall performance in the whole dataset. Moreover, the mAP achieved by LFNet-v2 under foggy scenes was 0.427, which was still significantly higher than the mAPs achieved by other comparison algorithms. For clear scenes, the mAP value of the algorithm proposed in this paper is 0.477, the third lowest of all algorithms, 0.026 and 0.006 lower than the mAP of CenterNet and STAC respectively. Furthermore, **Table 3** shows that the proposed method achieved the highest *AP*_50_, *AP*_75_, *AP_M_* and *AP_L_* in foggy scenarios and the highest *AP*_50_ and *AP_M_* in clear scenes. For images in the whole dataset, the proposed LFNet-v2 achieved the best performances in *AP*_50_, *AP*_75_, *AP_M_* and *mAP*, and the second best performances in *AP_L_*, which was 0.004 lower than the highest *AP_L_* obtained by the CenterNet. The *AP_S_* of LFNet-v2 ranked only third, 0.056 and 0.037 lower than the *AP_S_* achieved by SoftTeacher and CenterNet, respectively.

**Table 3:** Comparison of the proposed method with eight state-of-the-art object detection methods.

Networks		Faster-RCNN	CenterNet	YOLOx	DCNN	SIUM	DarkC-DCN	SoftTeacher	STAC	LFNet-v2
Foggy	AP_50_	0.228	0.541	0.512	0.289	0.368	0.392	0.563	0.42	0.592
	AP_75_	0.194	0.329	0.396	0.211	0.313	0.325	0.409	0.372	0.491
	AP*_S_*	0.091	0.212	0.205	0.101	0.137	0.136	0.241	0.181	0.195
	AP*_M_*	0.161	0.367	0.412	0.218	0.297	0.315	0.384	0.361	0.519
	AP*_L_*	0.187	0.414	0.407	0.268	0.314	0.305	0.414	0.413	0.501
	mAP	0.178	0.321	0.325	0.162	0.248	0.287	0.383	0.311	0.427
Clear	AP_50_	0.464	0.663	0.710	0.475	0.574	0.606	0.721	0.679	0.750
	AP_75_	0.308	0.511	0.588	0.365	0.421	0.427	0.521	0.478	0.563
	AP*_S_*	0.209	0.356	0.301	0.213	0.267	0.274	0.365	0.287	0.299
	AP*_M_*	0.357	0.565	0.548	0.364	0.439	0.447	0.590	0.493	0.605
	AP*_L_*	0.363	0.680	0.575	0.394	0.484	0.481	0.618	0.511	0.585
	mAP	0.270	0.503	0.469	0.352	0.406	0.385	0.471	0.483	0.477
Total	AP_50_	0.346	0.602	0.611	0.382	0.471	0.499	0.642	0.550	0.671
	AP_75_	0.251	0.420	0.492	0.288	0.367	0.376	0.465	0.425	0.527
	AP*_S_*	0.150	0.284	0.253	0.157	0.202	0.205	0.303	0.234	0.247
	AP*_M_*	0.259	0.466	0.480	0.291	0.368	0.381	0.487	0.427	0.562
	AP*_L_*	0.275	0.547	0.491	0.331	0.399	0.393	0.516	0.462	0.543
	mAP	0.224	0.412	0.397	0.257	0.327	0.336	0.427	0.397	0.452

### 4.4 Running times

The inference speed of LFNet-v2 presented in this article is shown in **Table 4**. In this experiment, the proposed method is compared with other algorithms in terms of model size and inference speed, and these algorithms include SSD (Liu et al., 2016), M2DET (Zhao et al., 2019), Faster R-CNN (Ren et al., 2015), YOLOv3 (Redmon and Farhadi, 2018), YOLOv4 (Bochkovskiy et al., 2020), YOLOv5 and LFNet (Shen et al., 2020). The sizes of all images input into the model were 416 * 416. The model’s inference speed of 34.30 FPS ranked third among all comparison algorithms. SSD and YOLOv3 had the highest inference speeds of 37.03 and 36.55 respectively. However, the inference speed of LFNet-v2 was only 7.37% and 6.15% lower than that of these two methods, respectively. Since SSD and YOLOv3 are classical real-time object detection algorithms, it can be inferred that the proposed LFNet-v2 can also perform real-time smoke detection tasks in forest scenes. In addition, the model size of LFNet-v2 is 21.5MB, which is much smaller than other algorithms. Specifically, the model size of LFNet-v2 is 90.8% smaller than that of YOLOv3. This suggests that the algorithm proposed in this paper is more suitable than other real-time object detection algorithms for devices with limited storage resources. Therefore, the proposed LFNet-v2 possess high practicality.

**Table 4:** Inference speed and model size.

Networks	SSD	M2DET	Faster RCNN	YOLOv3	YOLOv4	YOLOv5m	LFNet	LFNet-v2
Backbone	VGG16	VGG16	ResNet50	DarkNet53	CSPDarkNet53	CSPDarkNet53	–	–
FPS	37.03	11.96	8.06	36.35	21.42	27.27	27.92	34.30
Size	100 MB	238 MB	108 MB	236 MB	245 MB	81.7 MB	22.5 MB	21.5 MB

### 4.5 Ablation study

To demonstrate the practical value of the innovation proposed in this paper, we conduct ablation experiments by removing or replacing certain modules in this section.

#### 4.5.1 Smoke-aware consistency

**Figure 7:**
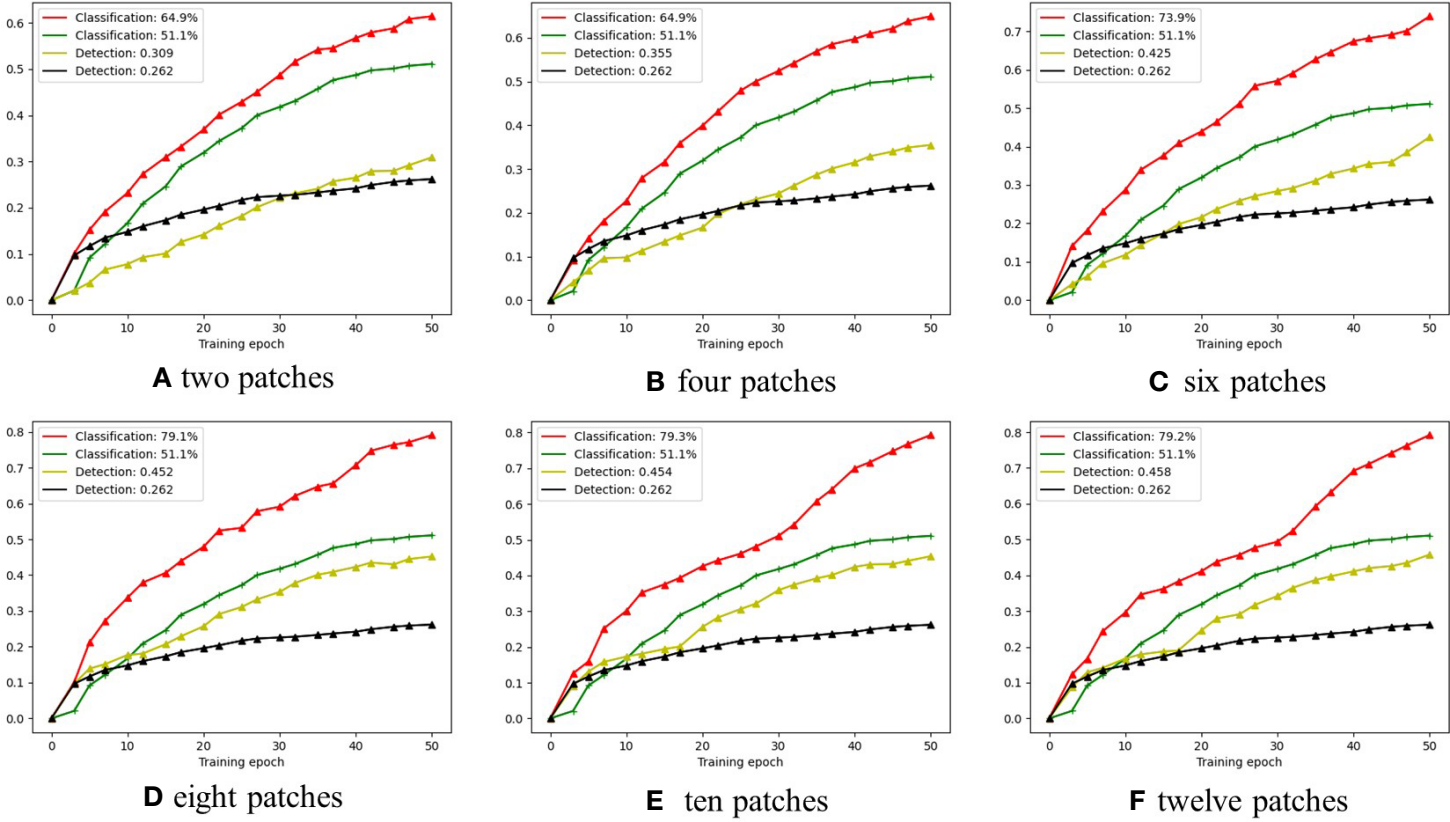
The smoke classification and detection accuracy when the proposed SAC adopts different numbers of image patches.

As shown in **Figure 7**, the red curves represent the classification performances achieved by LFNet-v2 with the SAC approach, while the green curves represent the classification performances achieved by LFNet-v2 without the SAC approach; the yellow curves represent the detection performances achieved by LFNet-v2 with the SAC approach, while the black curve represents the detection performances achieved by LFNet-v2 without the SAC.

From **Figures 7A–F** show the cases of two, four, six, eight, ten, and twelve patches cropped from the original images according to the pseudo labels, respectively. **Figure 7D** shows that for smoke classification and detection, the best strategy is to crop eight patches from the original images according to the pseudo labels. Specifically, when eight patches were cropped from the original image for SAC, the classification accuracy improved by 28% and the mAP improved by a value of 0.19 compared to the model without the SAC method. Classification and detection performance then did not improve significantly as more patches were cropped out of the original image. The main reason for this is that the eight patches cover eight directions centered on the pseudo label as shown in **Figure 4**. Therefore, cropping too many patches leads to redundancy of background information and does not significantly improve model performance. With the number of cropped patches still increasing, the performance impact of SAC will trend downwards.

#### 4.5.2 Different image augmentation strategies

**Table 5** illustrates the impact of low dimensional data augmentation on the wildfire smoke detection. As can be seen from **Table 5**, the model performs best when eight different data augmentation methods are used simultaneously. When only one data augmentation method was chosen, the model performed the worst, with mAP of only 0.427. In addition, we can see from the trend in the **Table 5** that the more methods of data augmentation that are used, the better the performance of the model are achieved.

**Table 5:** Ablation study for different data augmentation on SAC.

ID	GlassBlur	Histogram Equalization	Motion Blur	Gamma Contrast	Gaussian Noise	Average Blur	Fliplr	Snow	mAP
I	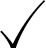								0.427
II	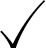	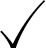							0.429
III	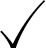	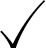	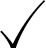						0.431
IV	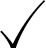	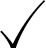	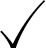	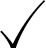					0.434
V	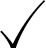	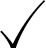	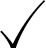	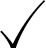	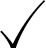				0.431
VI	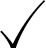	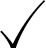	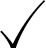	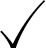	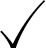	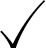			0.447
VII	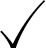	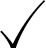	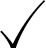	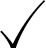	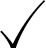	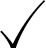	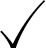		0.442
VIII	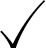	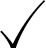	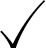	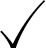	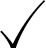	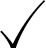	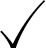	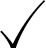	0.452

#### 4.5.3 Triple classification assistance

In this paper, we propose a triple classification assistance strategy to divide the forest smoke detection task into three categories: i.e., smoke, sky, and background. The results of the ablation experiments associated with it are shown in **Table 6**.

**Table 6:** Ablation study for triple classification assistance on eight state-of-the-art object detection networks.

Networks		Faster-RCNN	CenterNet	YOLOx	DCNN	SIUM	DarkC-DCN	SoftTeacher	STAC	LFNet-v2
	double	0.107	0.196	0.216	0.076	0.157	0.197	0.276	0.206	0.352
Foggy	triple	0.178	0.321	0.325	0.162	0.248	0.287	0.383	0.311	0.427
	double	0.211	0.405	0.381	0.272	0.287	0.277	0.397	0.381	0.411
Original	triple	0.270	0.503	0.469	0.352	0.406	0.385	0.471	0.483	0.477
	double	0.159	0.301	0.299	0.174	0.222	0.237	0.337	0.294	0.382
Total	triple	0.224	0.412	0.397	0.257	0.327	0.336	0.427	0.397	0.452
	difference	0.065	0.111	0.098	0.083	0.105	0.099	0.090	0.103	0.070

In **Table 6**, the double means that the scene is divided into background and smoke only, while the triple means that the scene is divided into background, smoke and sky. **Table 6** shows that triple classification assistance had the greatest impact on CenterNet, with its detection performance improved by 0.111, followed by SIUM (0.105) and STAC (0.103). In addition, the triple classification help also improved the mAP of LFNet-v2 by 0.07.

### 4.6 The effectiveness of smoke-aware consistency

In this subsection we analyze the practical effectiveness of the proposed SAC for smoke detection in forest scenes based on intuitive sensing effects.

#### 4.6.1 Adaptive ability for different background

**Figure 8:**
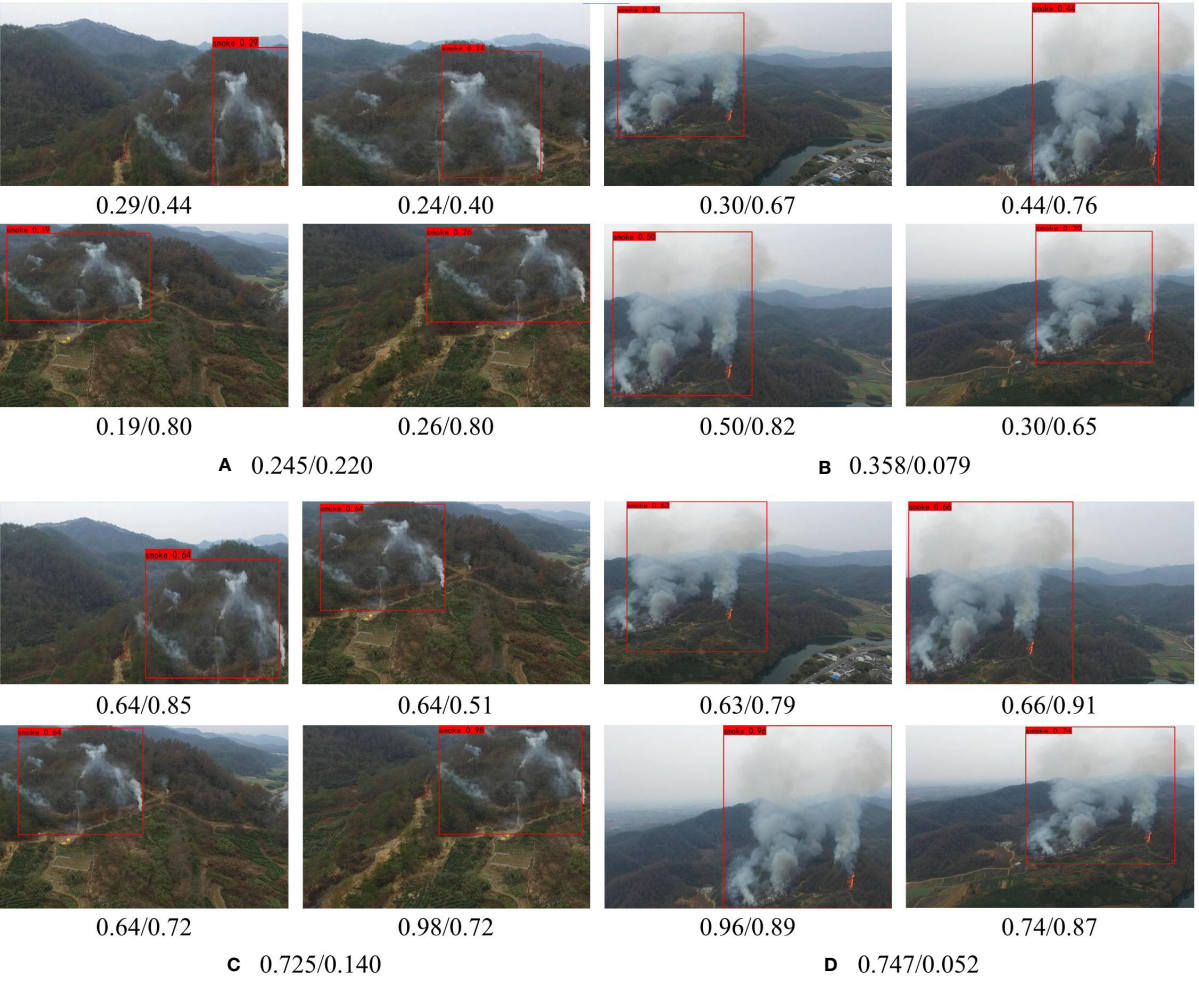
Ablation experiment on the adaptability of SAC to different backgrounds.

This subsection mainly focuses on the adaptability of LFNet-v2 to different backgrounds. As shown in **Figure 8**, the first and second rows of images are the detection results of LFNet-v2 without the SAC training strategy, while the third and fourth rows are the detection results of LFNet-v2 with the SAC training strategy. The front and back of/represent confidence and intersection over Union (IOU) respectively. The front and back of/in each captions for sub-image represent the mean value of confidence and IOU respectively. It can be observed from **Figure 8** that when SAC strategy is applied, LFNet-v2 can obtain better confidence, and the variance between IOU is smaller for different environmental backgrounds than the case when SAC is not used.

#### 4.6.2 Adaptive ability for different size

**Figure 9:**
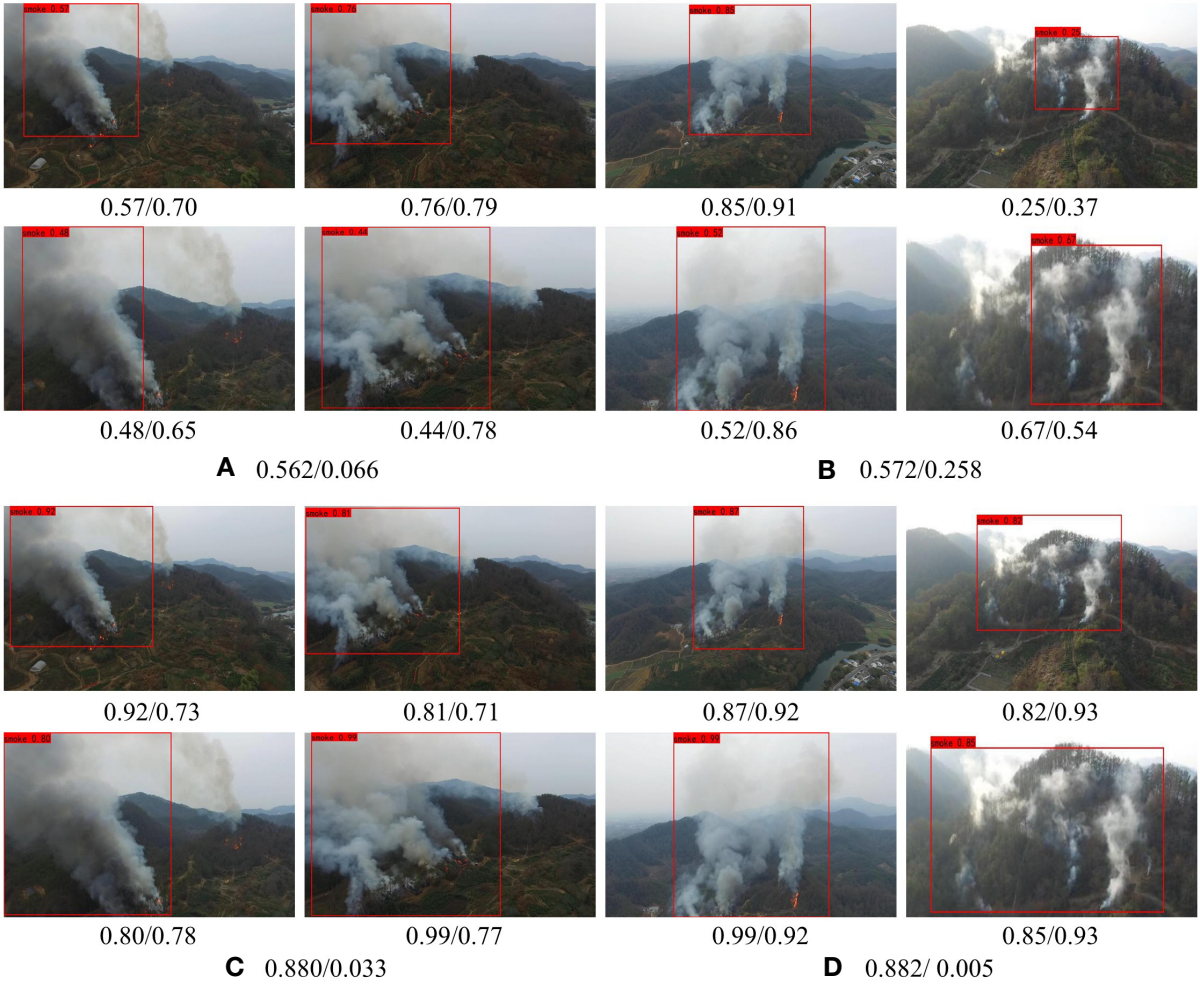
Ablation experiment on the adaptability of SAC to smoke with different size.

The influence of SAC on the performance of smoke image detection of different sizes is discussed in this paper. The first and second rows of **Figure 9** are the results of LFNet-v2 output without SAC training, while the third and fourth rows are the results of LFNet-v2 output with SAC training. Moreover, the images in the first row and the third row are the images of fires and smoke in the original forest scene, while the images from the second row and fourth row are those after our method was carried out on the smoke region. As shown in **Figure 9**, when using the SAC strategy in the model, LFNet-v2 could achieve higher confidence, which hardly affected the accuracy of the smoke.

#### 4.6.3 Anti-disturbance ability for different degradation

**Figure 10:**
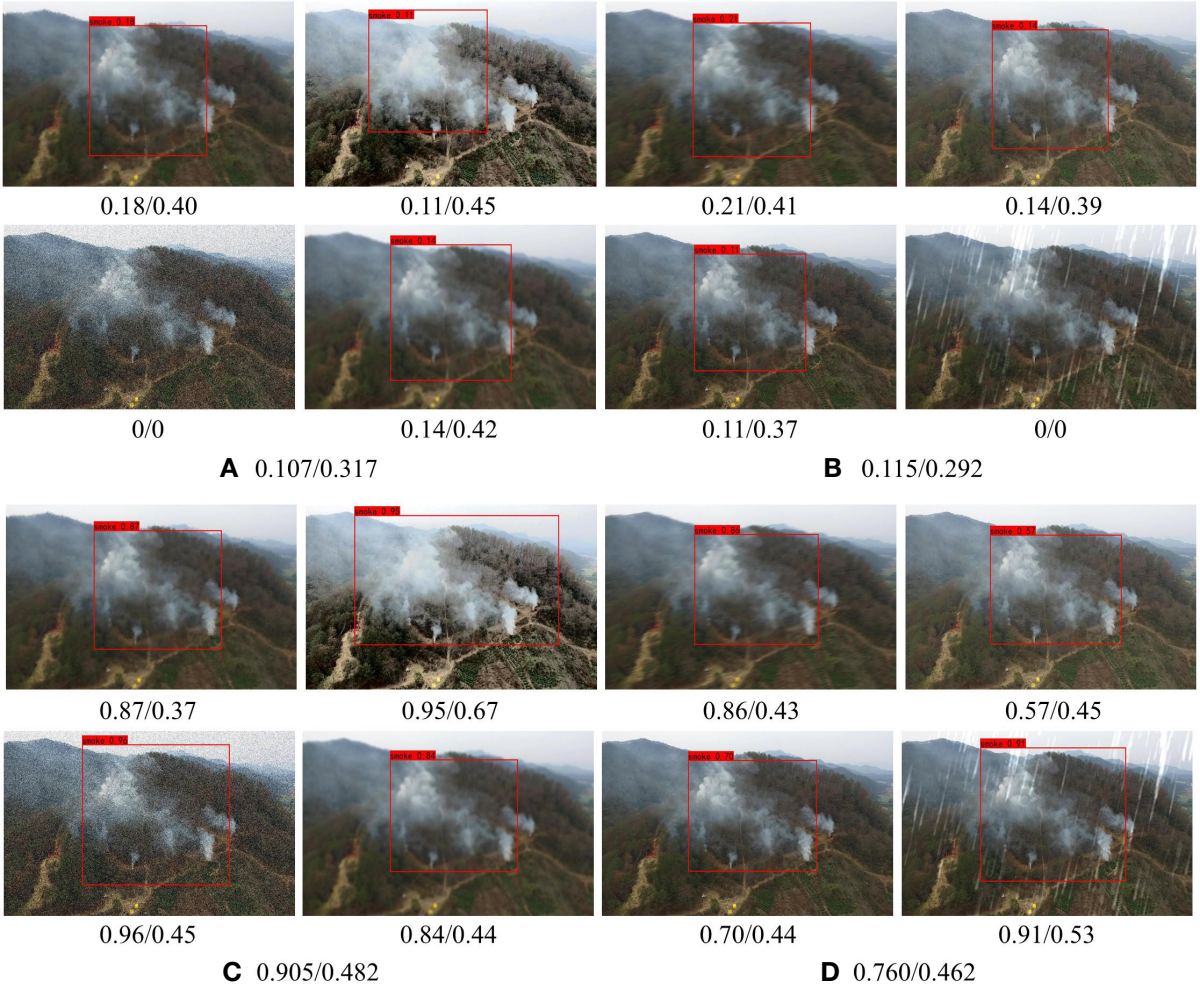
Ablation experiment of anti-disturbance ability of SAC to different degraded environments.

This subsection discusses how the detection capability of the model will change when it is exposed to different disturbances. As shown in **Figure 10**, it is obvious that when LFNet-v2 uses the SAC strategy, the detection ability of the model was significantly improved, both in terms of confidence and IOU. As can be seen from **Figure 10**, SAC increases the confidence of the model by 0.798 and 0.645 and increases the IOU by 0.165 and 0.170 respectively for the same scenario.

## 5 Conclusion

In this paper we propose a deep learning-based smoke detection model for forest scenes with smaller model size and faster inference. In addition, we also design a new semi-supervised training strategy, SAC strategy, which can improve the performance of the model against interference in different scenes and with different smoke sizes. The experimental results show that this method is better than other smoke detection algorithms in forest scenes.

However, the inference speed of the current model remains relatively slow. For this reason, in the future we will continue to optimize the model structure so that the computational complexity of the model enable real-time smoke detection on high-resolution images captured by drones (Alsamhi et al., 2021; Saif et al., 2021; Alsamhi et al., 2022; Alsamhi et al., 2022).

## Data availability statement

The original contributions presented in the study are publicly available. This data can be found here: https://github.com/CleanerWang/LFNet-v2.

## Author contributions

CW design of the experiments and wrote the first draft of the manuscript. AG and HF acquired the financial support for the project and contributed significantly to analysis and manuscript preparation. EG completed the experiment and sorted out the data. JH and ZS contributed to manuscript revision, read, and approved the submitted version.

## Funding

This research has been funded by the MINECO national project with reference (PID2019-106702RB-C21/AEI/10.13039/501100011033).

## Acknowledgments

The authors would like to thank the editor and reviewers for their constructive comments and valuable suggestions that greatly improve this work.

## Conflict of interest

The authors declare that the research was conducted in the absence of any commercial or financial relationships that could be construed as a potential conflict of interest.

## Publisher’s note

All claims expressed in this article are solely those of the authors and do not necessarily represent those of their affiliated organizations, or those of the publisher, the editors and the reviewers. Any product that may be evaluated in this article, or claim that may be made by its manufacturer, is not guaranteed or endorsed by the publisher.
